# Breast cancer research output, 1945-2008: a bibliometric and density-equalizing analysis

**DOI:** 10.1186/bcr2795

**Published:** 2010-12-22

**Authors:** Ronan W Glynn, Cristian Scutaru, Michael J Kerin, Karl J Sweeney

**Affiliations:** 1 Department of Surgery, Clinical Science Institute, National University of Ireland Galway, Costello Road, Galway, Ireland; 2 Department of Information Science, Charité-Universitätsmedizin Berlin, Free University Berlin, 12203 Berlin, Germany

## Abstract

**Introduction:**

Breast cancer is the most common form of cancer among women, with an estimated 194,280 new cases diagnosed in the United States in 2009 alone. The primary aim of this work was to provide an in-depth evaluation of research yield in breast cancer from 1945 to 2008, using large-scale data analysis, the employment of bibliometric indicators of production and quality, and density-equalizing mapping.

**Methods:**

Data were retrieved from the Web of Science (WOS) Science Citation Expanded database; this was searched using the Boolean operator, 'OR', with different terms related to breast cancer, including "breast cancer", "mammary ductal carcinoma" and "breast tumour". Data were then extracted from each file, transferred to Excel charts and visualised as diagrams. Mapping was performed as described by Groneberg-Kloft *et al*. in 2008.

**Results:**

A total of 180,126 breast cancer-associated items were produced over the study period; these had been cited 4,136,224 times. The United States returned the greatest level of output (*n *= 77,101), followed by the UK (*n *= 18,357) and Germany (*n *= 12,529). International cooperation peaked in 2008, with 3,127 entries produced as a result; relationships between the United States and other countries formed the basis for the 10 most common forms of bilateral cooperation. Publications from nations with high levels of international cooperation were associated with greater average citation rates. A total of 4,096 journals published at least one item on breast cancer, although the top 50 most prolific titles together accounted for over 43% (77,517/180,126) of the total output.

**Conclusions:**

Breast cancer-associated research output continues to increase annually. In an era when bibliometric indicators are increasingly being employed in performance assessment, these findings should provide useful information for those tasked with improving that performance.

## Introduction

In 2009, an estimated 194,280 new cases of breast cancer were diagnosed in the United States; breast cancer was estimated to account for 27% of all new cancer cases and 15% of cancer-related mortality in women [1]. Similarly, in Europe in 2008, the disease was reckoned to account for some 28% and 17% of new cancer cases and cancer-related mortality in women, respectively [2].

The last 50 years have seen an exponential increase in scientific yield generally, and particularly in oncology; a recent report demonstrated that in January of 2009 alone there were 11,215 new cancer-related papers and 1,220 review articles indexed in Pubmed [3]. The importance of quantitative and qualitative assessment of scientific output has increased in tandem with this information explosion, and these assessments now play an integral role in decisions regarding grant funding and prioritisation of resources, as exemplified by the Research Assessment Exercise in the UK [4]. Despite its aforementioned disease burden, relatively little effort has previously been made to understand the trends emanating from the breast cancer-associated literature. While there has been some concentration on the bibliometrics of cancer research generally [5,6], just three publications have evaluated breast-related output specifically; Dalpe *et al*. focused on the identification of BRCA1 and BRCA2 in the 1990 s [7], while Donato *et al*. published an analysis of the Portuguese contribution [8], and Li and McCain focused specifically on the development of research themes in the radiological detection of breast cancer [9]. The primary aim of this present work was thus to provide an in-depth evaluation of research yield in breast cancer from 1945 to 2008, using large-scale data analysis, the employment of bibliometric indicators of production and quality, and density-equalizing mapping.

## Materials and methods

### Data source

Data were retrieved from the Web of Science (WOS) Science Citation Expanded database (SCI-Expanded) produced by Thomson Reuters. In order to approximate the overall number of published items on breast cancer, the following search strategy was employed; TS = ((phyllodes tumo$r$) OR (Cystosarcoma Phyllo$des) OR (Malignant Cystosarcoma Phyllodes) OR (breast invasive ductal carcinoma) OR (infiltrating duct carcinoma$) OR (mammary ductal carcinoma$) OR (breast cancer) OR (breast neoplasm$) OR (breast tumo$r$) OR (human mammary neoplasm$) OR (human mammary carcinoma$)) where TS = Topic search, $ = any character. Because this work was designed to assess overall activity in relation to breast cancer, we did not refine our search to include some document types such as original articles or reviews, or to exclude others such as letters and editorials. The time span analysed was 1945 to 2008 inclusive. The search was performed in November 2009, and thus 2009 was excluded as database entries for this period would not have been complete at the time of the search.

Each item of information downloaded from the WOS was contained in a 'data block'. Each block was preceded by a tag which gave information about the content of the block (that is, AU = authors, TI = title, PY = publication year). Software developed at the Charite University in Berlin was then employed to parse the data. Each time it found a tag it read the associated data and saved it to an Access database; the information was then later transferred to an Excel database for analysis. Published items were analysed using the citation report method as described previously [10,11]. The number of citations per year and the average number of citations per item were assessed, thereby indicating the average number of citing articles for all items in the set. This is the sum of the times cited divided by the number of results found.

Mapping was performed as described by Groneberg-Kloft *et al*. in 2008 [12]. Those nations which had contributed output were resized according to one of a number of different variables under study; that is, the average number of citations per item from each country. As part of this resizing procedure, the area of each country was scaled relative to, for example, the total number of items it had published on breast cancer. Specific calculations were based on Gastner and Newman's algorithm [13], published in 2004. These calculations employ a diffusion equation in the Fourier domain borrowed from elementary physics, which allows variable resolution by tracking moving boundaries [13,14].

Cooperation analysis was employed to determine bilateral and multilateral cooperation between countries on breast cancer research. A cooperation network between countries was computed by checking all combinations of those countries which registered international cooperation on at least 25 items over the study period. These data were then saved to a "matrix" or two-dimensional table, and the software then read this matrix and produced a density-equalising map which graphically represented this data. The threshold of 25 articles was set to improve readability.

Journals which had published on breast cancer were analysed relative to both the Journal Impact Factor (IF) and the recently developed Eigenfactor (EF). The former is based on two elements; the numerator, which is the number of citations in the current year to items published in the previous two years, and the denominator, which is the number of substantive articles and reviews published in the same two years [15]. The EF is calculated based on a complex algorithm that takes into account not only the quantity of citations but also their "quality" by assigning weights to the source of the citations. The full details of the algorithm can be found online [16].

## Results

### Total number of published items

**Figure 1:**
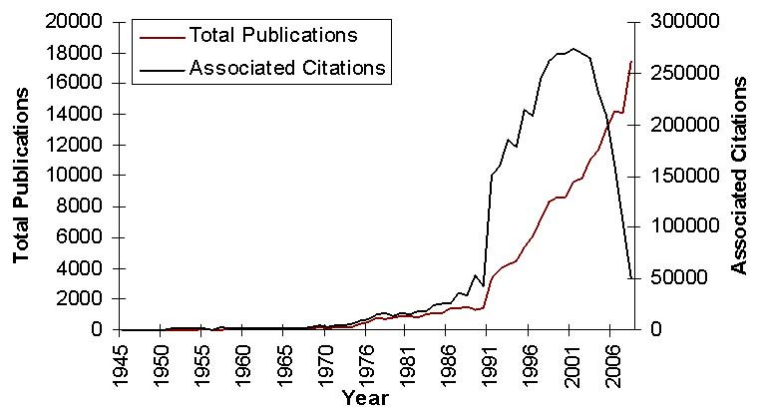
**Total breast cancer-related output and associated citations, Web of Science, Science Citation Expanded Database, 1945-2008**.

The number of published items on breast cancer was employed as an index of research productivity. During the period 1945 to 2008 (1974 excluded, *n *= 352), a total of 180,126 items were produced on this topic, as catalogued in the WOS. The earliest studies catalogued were published in 1945 (*n *= 17), although it was 1990 before activity began to increase considerably, year on year (Figure 1); output more than doubled from 1990 (*n *= 1,436) to 1992 (*n *= 3,342). The greatest output for any year was that for 2008 (*n *= 17,413).

### Total number of citations

The 180,126 indexed items have been cited 4,136,224 times since 1945. Figure 1 demonstrates the parallel increase in the number of citations in conjunction with the increase in published items. Articles published in 2001 were responsible for more citations than those published in any other year (*n *= 274,601). The average number of citations per item was greatest in 1957, however, when 40 items were responsible for 2,767 citations, returning an average of 69.01 citations per item published. There has been a downward trend in the average number of citations per item since the millennium.

### Country of origin

**Figure 2:**
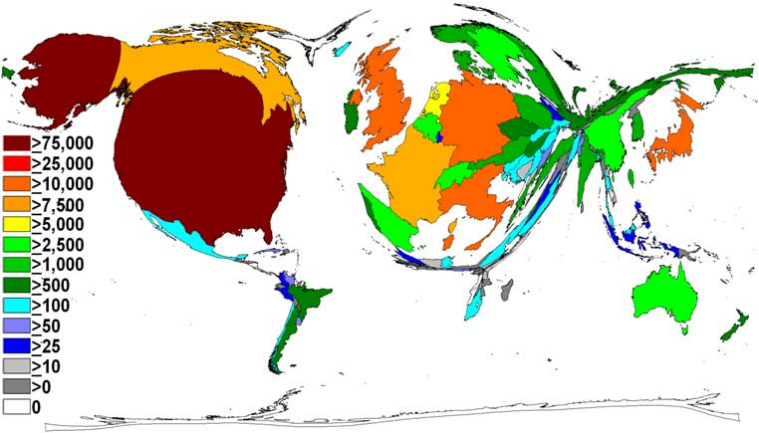
**Density equalizing calculations, total output by country**. Illustration of the total number of breast-related items, per country. The size of each country has been scaled in proportion to the total number of publications. A colour-coded system shows the publication numbers.

**Figure 3:**
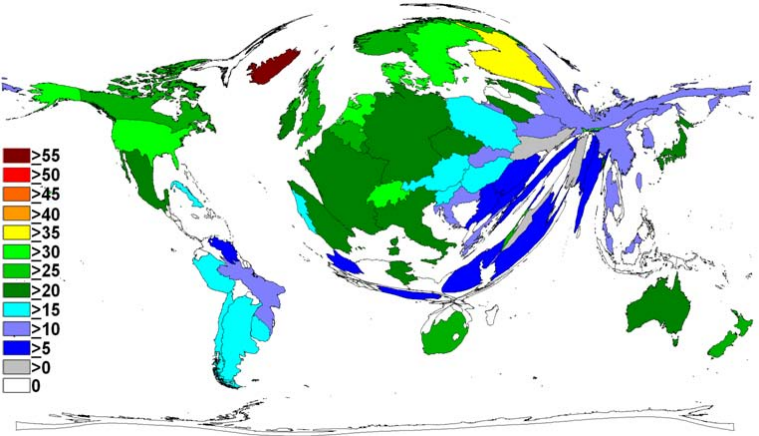
**Density equalizing calculations, research quality**. Illustration of the average number of citations per breast cancer-related item, per country. The size of each country has been scaled in proportion to the average number of citations per item. A colour-coded system shows the average number of citations per item. Threshold excludes countries with <30 items published.

A total of 155 different countries contributed to the literature on breast cancer over the study period. The United States was responsible for the greatest output, returning 77,101 items. Other high output countries included the United Kingdom (*n *= 18,357), Germany (*n *= 12,529), Italy (*n *= 10,828) and Japan (10,109) (Table 1). Density equalising mapping of this dataset demonstrates that a relatively small number of countries was responsible for the majority of the output (Figure 2). The Gambia had the highest average citation rate per item (67.67), followed by Kenya (40.69), and Costa Rica (39.53) (Table 1). When confined to those countries which had produced at least 30 items, however, those with the highest average citation per item were Iceland (56.62), Finland (35.48), Denmark (32.88) and Switzerland (31.85) (Figure 3).

**Table 1:** Leading countries by output and average citations per item, 1945-2008

	Top countries - output			Top countries - average citings per item			
		
Rank	Country	No. Publications	Times Cited	Country	No. Publications	Times Cited	Average Citings/Item
1	United States	77,101	2,389,337	Gambia	3	203	67.67
2	UK	18,357	484,550	Kenya	16	651	40.69
3	Germany	12,529	256,883	Costa Rica	19	751	39.53
4	Italy	10,828	227,078	Finland	2,334	82,802	35.48
5	Japan	10,109	205605	Denmark	2,377	78,163	32.88
6	France	9,412	235,248	Switzerland	2,989	95,201	31.85
7	Canada	9,002	266,803	Netherlands	5,594	173,652	31.04
8	Netherlands	5,594	173,652	United States	77,101	2,389,337	30.99
9	Australia	4,531	110,100	Sweden	4,102	125,209	30.52
10	Sweden	4,102	125,209	Canada	9,002	266,803	29.64
11	Spain	3,680	73,845	Norway	1,884	55,816	29.63
12	China	3,593	42,761	Haiti	1	29	29.00
13	Switzerland	2,989	95,201	Philippines	28	772	27.57
14	Belgium	2,636	66,095	Uganda	9	247	27.44
15	Denmark	2,377	78,163	UK	18,357	484,550	26.40
16	Finland	2,334	82,802	South Africa	475	12208	25.70
17	Austria	2,136	38,079	New Zealand	662	16,820	25.41
18	South Korea	2,118	25,614	Israel	2,086	52,416	25.13
19	Israel	2,086	52,416	Belgium	2,636	66,095	25.07
20	Greece	1,947	28,110	Rwanda	1	25	25.00
21	Norway	1,884	55,816	France	9,412	235,248	24.99
22	Taiwan	1,645	23,297	Senegal	6	147	24.50
23	India	1,347	11,437	Australia	4,531	110,100	24.30
24	Poland	1,341	21,408	Panama	11	266	24.18
25	Turkey	1,297	8,240	Botswana	1	24	24.00
26	Brazil	969	10,651	Paraguay	1	24	24.00
27	Russia	968	13,507	Mozambique	1	23	23.00
28	Ireland	876	18,979	Tunisia	103	2,321	22.53
29	Singapore	693	13,607	Colombia	27	606	22.44
30	Argentina	597	11,652	Estonia	62	1,356	21.87

**Figure 4:**
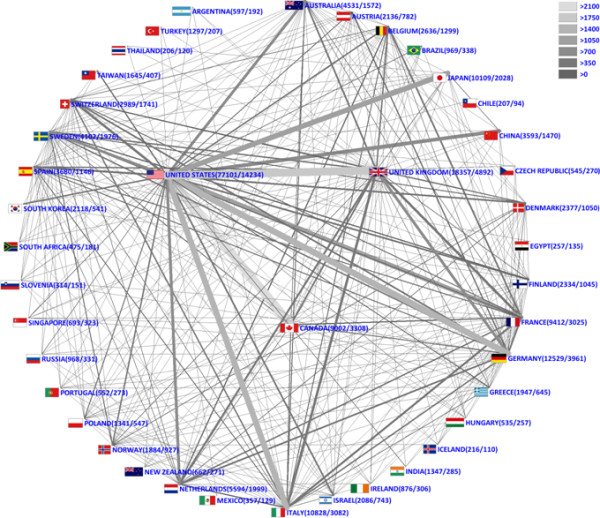
**Radar chart of cooperation density**. Threshold >25 international cooperative partnerships.

Cooperation analysis was employed to assess bilateral and multilateral cooperation from 1973 to 2008; the first item in the dataset produced as a result of international cooperation was published in 1973. In total, 142 different countries had collaborated on at least one item published. International cooperation increased steadily through the study period, reaching a peak in 2008, with 3,127 entries produced as a result of cooperation. Bilateral cooperation was the most common form of cooperation (19,437 entries), followed by trilateral cooperation (*n *= 3,157) and quadrilateral cooperation (*n *= 836). Cooperation between the United States and Canada was the most common form of bilateral cooperation (*n *= 2,223), followed by that between the United States and the United Kingdom (*n *= 2,007) (Figure 4). Relationships between the United States and other countries formed the basis for the 10 most common forms of bilateral cooperation (Table 2).

**Table 2:** Top 25 collaborating relationships, breast cancer-related items, 1949-2008

Rank	Country 1	Country 2	No. Publications
1	Canada	United States	2,223
2	United Kingdom	United States	2,007
3	Germany	United States	1,601
4	Italy	United States	1,460
5	Japan	United States	1,294
6	France	United States	1,129
7	China	United States	953
8	Netherlands	United States	743
9	Sweden	United States	740
10	Australia	United States	723
11	Germany	United Kingdom	719
12	Switzerland	United States	668
13	France	United Kingdom	639
14	Italy	United Kingdom	638
15	Spain	United States	559
16	Netherlands	United Kingdom	548
17	France	Italy	541
18	Israel	United States	482
19	Australia	United Kingdom	459
20	Germany	Netherlands	450
21	France	Germany	447
22	Denmark	United States	442
23	Germany	Switzerland	439
24	Canada	United Kingdom	437
25	Finland	United States	433

No. Publications, the number of items published as a result of cooperation between the two countries listed.

### Publishing journals

A total of 4,096 journals had published at least one item on breast cancer. The journals which have published most prolifically on breast cancer, led by *Cancer Research *(5,290 items), are listed in Table 3. The top 50 most prolific titles, representing just 1.2% of all contributing journals, together accounted for over 43% (77517/180126) of the total output. Thirty of these top 50 titles were in the category 'Oncology' of the Journal Citation Report; other represented subject categories included 'Surgery' (*n *= 5), 'Pathology' (*n *= 4), 'Radiology, Nuclear Medicine and Medical Imaging' (*n *= 4). 'Biochemistry and Molecular Biology' (*n *= 3), and 'Medicine, General and Internal' (*n *= 3). The median impact factor (IF) and Eigenfactor (EF) of these titles was 4.73 and 0.05, respectively. *Cancer Research *also recorded the highest number of citations overall (*n *= 309,568), followed by the *Journal of Clinical Oncology *(*n *= 177,189), *Cancer *(*n *= 166,834), the *Journal of the National Cancer Institute (JNCI) *(*n *= 131,637), and the *British Journal of Cancer *(*n *= 110,307) (Table 3).

**Table 3:** Leading titles, breast cancer-related items, 1945 to 2008

Top 50 journals by output							
**Journal**	**IP**	**IF**	**EF**	**Journal**	**IP**	**IF**	**EF**

Cancer Res	5290	7.51	0.43	J Pathol	830	5.12	0.04
Cancer	4542	5.24	0.12	Carcinogenesis	821	4.93	0.05
Breast Cancer Res Tr	4210	5.68	0.03	Gynecol Oncol	800	2.92	0.04
Brit J Cancer	4075	4.85	0.09	B Cancer	794	1.09	0.00
J Clin Oncol	4003	17.16	0.35	Biochem Biophys R Co	792	2.65	0.24
Eur J Cancer	3503	4.48	0.05	Breast Cancer Res	780	5.05	0.02
Int J Cancer	3370	4.73	0.11	J Nucl Med	769	6.66	0.05
Clin Cancer Res	2746	6.49	0.22	EJSO	726	2.49	0.01
Anticancer Res	2298	1.39	0.03	Semin Oncol	714	3.96	0.02
JNCI	2286	14.93	0.10	J Surg Oncol	713	2.48	0.01
Oncogene	2125	7.22	0.26	Cancer Chemoth Pharm	650	2.74	0.01
Ann Oncol	2012	4.94	0.05	Geburtsh Frauenheilk	649	0.35	0.00
J Biol Chem	1703	5.52	1.33	Am J Surg	630	2.61	0.03
Lancet	1692	28.41	0.41	Am J Pathol	628	5.70	0.10
Int J Radiat Oncol	1509	4.64	0.07	Endocrinology	619	4.95	0.12
Cancer Epidem Biom	1390	4.77	0.06	Am J Roentgen	614	2.94	0.05
Int J Oncol	1371	2.23	0.03				
				
Am J Epidemiol	1335	5.45	0.08	**Top 15 journals - by citation count**			
				
Ann Surg Oncol	1261	3.90	0.03	**Journal**	**IP**	**Citations**	**AC/I**
				
Cancer Lett	1206	3.50	0.05	Cancer Res	5290	309568	58.52
Eur J Cancer Suppl	1190	3.41	0.00	J Clin Oncol	4003	177189	44.26
Oncol Rep	1148	1.52	0.02	Cancer	4542	166834	36.73
Radiology	1127	6.00	0.09	JNCI	2286	131637	57.58
Breast	1068	2.16	0.01	Brit J Cancer	4075	110307	27.07
N Engl J Med	1048	50.02	0.68	Int J Cancer	3370	96406	28.61
J Steroid Biochem	1031	2.83	0.02	J Biol Chem	1703	93637	54.98
Modern Pathol	1010	4.68	0.03	Oncogene	2125	92621	43.59
Proc Amer Ass Cancer Re	976	-	-	PNAS	895	91620	102.37
Lab Invest	966	4.58	0.02	NEJM	1048	86248	82.30
Brit Med J	913	12.83	0.16	Clin Canc Res	2746	81183	29.56
JAMA	904	1.77	0.01	Lancet	1692	70743	41.81
Psycho-Oncol	902	3.15	0.01	Breast Cancer Res Tr	4210	57714	13.71
PNAS	895	9.38	1.70	Science	195	57017	292.39
Brit J Surg	883	4.92	0.03	JAMA	904	52380	57.94

IP, items published; IF, impact factor; EF, eigenfactor. IF and EF are taken from Thomson Reuters JCR, 2008.

## Discussion

In his seminal work on the exponential growth of science, *Little Science, Big Science*, Price noted in 1963 that all of the scientific periodicals founded since the first, the Journal de Scavaus (first published in 1665), had together produced a world total of six million scientific papers over the course of the preceding 300 years [17]. By contrast, Druss demonstrated that in just 23 years, from 1978 to 2001, a total of 8.1 million articles were published in Medline [18]. The results of this present analysis have demonstrated this growth in breast cancer research specifically, with an average 15% increase in output annually since 1945, and a greater than 100% increase since the millennium alone. This compares with a recent analysis of total scientific output from PubMed, which estimated an average growth rate of 4% per year between 1957 and 2007 [4].

This analysis has employed the citation count as a proxy measure of research quality. Forming an essential component in the dialogue of medical research [19], citations are regarded as a key indicator of the relevance and importance of a published item. We have shown a parallel increase in citation count with the number of breast cancer-related articles, a not unexpected finding recently mirrored in analyses of scientific output on scoliosis [20] and asthma [10]. The average number of citations per year was highest in 1957, although this was thanks largely to the citation classic by Bloom and Richardson in which they outlined their system for the histological grading of breast cancer and its association with prognosis [21]; it has since been cited 2,259 times. To put this figure into perspective, Garfield noted in 2006 that of 38 million items cited from 1900 to 2005, only 0.5% were cited more than 200 times [15]. Although there has been a decreasing trend in the average number of citations per item since the mid-1990 s, it is difficult to draw firm conclusions on the relevance of this finding; it may be explained by the sharp increase in the number of outputs in the intervening years, or indeed by the time-lag associated with citation analysis which results in an inherent bias towards older publications.

This analysis has demonstrated the leading role which the United States plays in breast cancer research, a finding previously noted in other scientific disciplines [22,23]. This is not surprising given the enormous amount of money spent on the management of breast cancer there annually; it has been estimated that new cases of breast cancer diagnosed globally in 2009 alone will have cost an estimated $28 billion; of this $28 billion, $16 billion was spent in the United States [24]. In addition to being the single largest contributor to the literature on breast cancer, the United States has further played a key role in fostering international cooperation, in particular with its neighbour Canada, but also with many European nations, including Germany, the United Kingdom and Italy.

The large number of nations involved in breast cancer research reflects its global burden. That said, the map of global production shown in Figure 2 clearly demonstrates the dramatic underrepresentation of South America, Africa, and to a lesser extent, Asia. Given that the majority of the predicted 26% increase in the incidence of breast cancer by 2020 will occur in the developing world [24], there needs to be a concerted effort to further involve these areas in future research initiatives, particularly focusing on how the cost-effective diagnosis and management of breast cancer can be delivered with levels of efficacy similar to those presently seen in Europe and the United States.

The quality of breast-related output from both the United States and the United Kingdom was high as measured using the average citation rate per published item as a proxy measure for quality. In addition, the contribution of many smaller countries, including Iceland, Finland, Switzerland and Denmark, was of high quality, with all four associated with impressive average citation rates. Interestingly, all of these countries collaborated internationally in a high proportion of their output (Figure 4) (Iceland 110/216, 50.92%; Finland 1,045/2,334, 44.77%; Switzerland 1,741/2,989, 58.24%; Denmark 1,050/2,377, 44.17%), suggesting perhaps that this form of cooperation results in improved quality, and hence citation rate, of associated output.

Our finding that the breast cancer-associated research has been published across over 4,000 journals reiterates the view that it is now impossible for those working in breast cancer to ensure that they appraise all of the relevant literature. Our work has, however, identified a core set of journals publishing on breast cancer, with the top 50 accounting for 43% of the total output. The median IF and EF of these titles compares particularly well with the median values for all 143 journals in the JCR category oncology in 2008 (2.66, 0.01, respectively), and alludes to the quality of output in this subject area.

There are a number of limitations to this work. Output from 1974 (*n *= 352, 0.2% of total output) was accidentally excluded during data collection, and hence, was not included in the subsequent analysis. In addition, this study has focused on entries contained in the Web of Science only, and it should be noted that the employment of other databases including PubMed and Scopus may have yielded slightly different results. That said, Web of Science covers the oldest publications with archived records back to 1900 [25], and should provide an accurate overview of output over the entire study period. Finally, while we have provided an overview of geographic output on breast cancer, we have not related our findings to underlying socio-economic and demographic variables, and clearly this would be an interesting future avenue for investigation.

## Conclusions

This work represents the first bibliometric assessment of research quantity and quality in breast cancer-associated literature. The results have demonstrated the ongoing expansion of that literature, while also identifying the key nations and journals involved in its production over the past half-century. In an era when bibliometric indicators are increasingly being employed in the assessment of individual, institutional and national performance, these findings should provide useful information for those tasked with improving that performance.

## Abbreviations

EF: Eigenfactor; IF: Journal Impact Factor; UK: United Kingdom; WOS: Web of Science.

## Competing interests

The authors declare that they have no competing interests.

## Authors' contributions

All four authors have made substantial contributions to the conception and design, acquisition, analysis and interpretation of the data in this study. All have also been involved in drafting the manuscript or revising it critically for important intellectual content and all have given final approval of the version to be published.
